# Bone Morphogenic Protein 2-Loaded Porous Silicon Carriers for Osteoinductive Implants

**DOI:** 10.3390/pharmaceutics11110602

**Published:** 2019-11-12

**Authors:** Michal Rosenberg, Dekel Shilo, Leonid Galperin, Tal Capucha, Karim Tarabieh, Adi Rachmiel, Ester Segal

**Affiliations:** 1 Department of Biotechnology and Food Engineering, Technion—Israel Institute of Technology, Haifa 3200003, Israel; michirosros@gmail.com (M.R.); leonidgal@campus.technion.ac.il (L.G.); 2 Department of Oral and Maxillofacial Surgery, Rambam Health Care Campus, Haifa 3109601, Israel; D_shilo@rambam.health.gov.il (D.S.); capuchatal@gmail.com (T.C.); karim.t@campus.technion.ac.il (K.T.); a_rachmiel@rambam.health.gov.il (A.R.); 3 Bruce Rappaport Faculty of Medicine, Technion—Israel Institute of Technology, Haifa 3109601, Israel; 4 Russell Berrie Nanotechnology Institute, Technion—Israel Institute of Technology, Haifa 3200003, Israel

**Keywords:** bone morphogenic protein 2, porous silicon, bone marrow mesenchymal stem cells, differentiation, osteoinduction, implants

## Abstract

Bone morphogenetic proteins (BMPs) are probably the most important growth factors in bone formation and healing. However, the utilization of BMPs in clinical applications is mainly limited due to the protein poor solubility at physiological pH, rapid clearance and relatively short biological half-life. Herein, we develop degradable porous silicon (PSi)-based carriers for sustained delivery of BMP-2. Two different loading approaches are examined, physical adsorption and covalent conjugation, and their effect on the protein loading and release rate is thoroughly studied. The entrapment of the protein within the PSi nanostructures preserved its bioactivity for inducing osteogenic differentiation of rabbit bone marrow mesenchymal stems cells (BM-MSCs). BM-MSCs cultured with the BMP-2 loaded PSi carriers exhibit a relatively high alkaline phosphatase (ALP) activity. We also demonstrate that exposure of MSCs to empty PSi (no protein) carriers generates some extent of differentiation due to the ability of the carrier’s degradation products to induce osteoblast differentiation. Finally, we demonstrate the integration of these promising BMP-2 carriers within a 3D-printed patient-specific implant, constructed of poly(caprolactone) (PCL), as a potential bone graft for critical size bone defects.

## 1. Introduction

Bone morphogenetic proteins (BMPs) are a well-studied family of osteoinductive growth factors which are responsible for bone formation during embryogenesis, bone remodeling, and bone regeneration [1,2,3]. BMP-2 and BMP-7 have been approved by the US Food and Drug Administration (FDA) for treatment of acute and open fractures of the tibial shaft and for oral maxillofacial surgeries [1,2,3]. For the latter, FDA-approved INFUSE^®^ bone grafts, which consists of recombinant human BMP-2 placed on an absorbable collagen sponge, are clinically-used to induce new bone tissue at the site of implantation [2]. Yet, the wide-spread use of BMPs for medical and dental applications is still limited due to its poor solubility at physiological pH [2,4,5,6], rapid clearance and relatively short biological half-life [2,7]. In addition, BMP-2 was found to induce ectopic bone formation, haematomas in soft tissues, and bone resorption around implants, and thus it is important to localize the protein to the required compartment in order to minimize these complications [8,9,10]. Therefore, a substantial research effort is focused on developing delivery systems for BMP-2 that will allow for controlled spatiotemporal release which would lower the administered dose, localize the protein delivery strictly to the defect region, prolong its retention time at the site of action, and maintain its stability. The majority of BMP-2 delivery systems are based on synthetic or natural polymers [8,11]. For the latter (e.g., collagen), their biodegradation is difficult to control, resulting in undefined release kinetics of the entrapped protein [12]. As for synthetic polymers, their acidic degradation products may locally reduce the pH and induce inflammation at the implant site. In addition to this acidification, tendency to hydrophobicity which is typical for many polymers, such as poly(lactic-*co*-glycolic acid), may compromise the protein stability [13]. Other drawbacks encountered with the use of some synthetic polymers include retarded clearance rate, lack of biological function and chronic inflammation associated with high molecular weight polymers [14,15,16]. Metal scaffolds made of titanium are commonly used as support scaffolds for bone repair applications [17,18]. However, BMP molecules incorporated into titanium support scaffolds are either adsorbed to the surfaces or are superficially entrapped and therefore can be rapidly released in vivo [15].

Porous silicon (PSi) has been extensively studied in recent years for drug delivery applications [19,20,21] owing to its high surface area and porous volume, combined with its biocompatibility, as well as its ability to degrade into non-toxic products under physiological conditions [22,23]. In addition, PSi has been shown to exhibit beneficial features in the field of bone regeneration [24,25,26,27,28,29], including its ability to induce hydroxyapatite growth [30,31]. PSi has been shown to promote osteoblast adherence and initiate maturation process [32]. Furthermore, its degradation product, orthosilicic acid, has been shown to induce osteodifferntiation of mesenchymal stem cells (MSCs) into osteoblasts in vitro [33]. 

Many studies have demonstrated the potential of PSi-based delivery systems for protein therapeutics [34,35,36,37,38,39]. Loading of proteins into PSi carriers can be carried out at low temperatures without the use of strong organic solvents, which is advantageous when dealing with these sensitive macromolecules [23,40,41]. Moreover, proteins can be loaded via simple electrostatic adsorption which is optimal for persevering their delicate tertiary structure. The process of protein loading into PSi depends on the properties of the porous matrix (e.g., pore size and surface chemistry), as well as those of the protein (e.g., molecular size and structure, charge and hydrophilicity/hydrophobicity). Of importance are also properties of the loading solvent in terms of pH, ionic strength and composition [36,42,43,44,45]. The surface chemistry of the PSi exerts a major effect on the loading efficacy and can be easily tuned in order to better control the interactions between the protein molecules and the porous scaffold. These interactions subsequently dictate the structure of the adsorbed protein molecules and their bioactivity [36,37,46]. 

The present work aims to employ PSi nanostructures as carriers for BMP-2, allowing for the protein’s sustained release while retaining its biological activity. Two different loading approaches are examined, physical adsorption and covalent conjugation, and their effect on the loading and release kinetics is thoroughly studied. The fabricated carriers exhibit sustained release of the growth factor over a period of 35 days. The biological activity of the released BMP-2 was studied by characterizing its ability to stimulate osteogenic differentiation of bone marrow mesenchymal stem cells (BM-MSC). The degree of differentiation was examined by measuring the alkaline phosphatase (ALP) activity of MSCs after two weeks of exposure to the BMP-2 loaded PSi carriers. BM-MSCs cultured with BMP-2 loaded PSi carriers (via both physical adsorption and covalent attachment) exhibit relatively high ALP activity. Remarkably, empty PSi (no BMP-2) carriers have also demonstrated some degree of osteogenic differentiation, exhibiting an ALP activity which was significantly higher than that of the control untreated MSCs. After proving the therapeutic efficacy of this delivery system in vitro, we provide a glimpse to our current work, which focuses on the integration of these promising BMP-2 carriers within a 3D-printed patient-specific implant, constructed of poly(caprolactone) (PCL), as a bone graft for critical size bone defects. The latter affect more than 1.5 million people in the U.S. each year [47]. The loss or dysfunction of skeletal tissue due to trauma, ablative surgery, aging or diseases, can result in significant morbidity, as well as reduced quality of life. Current treatment strategies usually involve the use of autogenous bone grafts such as fibula, iliac crest and rib grafts as non-vascularized or as vascularized grafts. These grafts suffer from severe limitations including substantial challenging surgery, limited success, resorption of bone, donor-site morbidity, severe infections and residual pain [48,49,50]. Thus, there is an urgent need for developing new reliable bone regeneration strategies.

## 2. Materials and Methods 

### 2.1. Fabrication, Chemical Modification and Chracterization of PSiO_2_ Carriers

PSi was fabricated by anodization of heavily p-doped Si wafers (0.95 mΩ × cm resistivity, <100>-oriented, B-doped, purchased from Sil′tronix Corp., Archamps, France). First, the Si wafers were thermally oxidized at 400 °C for 2 h in ambient air in a tube furnace (Thermo Scientific, Lindberg/Blue M™ 1200 °C Split-Hinge, Waltham, MA, USA), followed by incubation in a solution of aqueous hydrofluoric acid (HF) (48%, Merck, Darmstadt, Germany), double-distilled water (ddH_2_O) and ethanol (99.9%, Merck, Darmstadt, Germany) (1:1:3 *v*/*v*) for 5 min. Subsequently, the samples were anodized in a two-step process (the anodization setup details were previously reported [51]). The first stage included anodization at a constant current density of 250 mA cm^™2^ for 30 s in a solution of aqueous HF and ethanol at a ratio of 3:1 (*v*/*v*). The resulting PSi layer was dissolved in an aqueous NaOH solution (0.1 M, Sigma-Aldrich Chemicals, Rehovot, Israel). The second stage included anodization at 250 mA cm^™2^ for 20 s. After each anodization step, the silicon surface was thoroughly washed with ethanol and dried under a nitrogen stream. Following anodization, the resulting PSi film was thermally oxidized at 800 °C for 1 h in ambient air, producing a porous SiO_2_ (PSiO_2_) scaffold. Next, a dicing saw (DAD3500; Disco, Tokyo, Japan) was used to cut the samples into 7 mm × 3 mm rectangles. Before dicing, the samples were spin coated with AZ4533 photoresist (MetalChem, Lod, Israel) at 4000 rpm for 1 min, followed by baking at 90 °C for 2 min. The diced samples were then soaked in acetone (Gadot, Haifa, Israel) for 3 h to remove the photoresist coating, thoroughly washed with ethanol and dried under a nitrogen stream. The physical properties of the neat PSiO_2_ samples (i.e., pore size and porous layer thickness) were studied by a Carl Zeiss Ultra Plus high-resolution scanning electron microscope (HR-SEM) at an accelerating voltage of 1 kV. The porosity of the PSiO_2_ was measured by gravimetry and spectroscopic liquid infiltration method (SLIM), as previously reported [51]. 

The chemically-modified PSiO_2_ carriers were prepared as follows: First, PSiO_2_ samples were amino-silanized by incubation in a solution of 1% *v*/*v* (3-Aminopropyl)triethoxysilane (APTES) in ddH_2_O and 1% *v*/*v N*-*N*-Diisopropylethylamine (DIEA) for 30 min, followed by baking at 100 °C for 15 min. Second, the amine-modified PSiO_2_ was incubated in a solution of succinic anhydride (10 mg mL^−1^) in acetonitrile and 2% *v*/*v* DIEA for 3 h. After which, the surface was thoroughly rinsed for several times with acetonitrile and ddH_2_O. Next, the samples were reacted with (ethyl-3-(3-(dimethylamino)propyl)carbodiimide) (EDC) (10 mg mL^−1^) and (*N*-hydroxysulfosuccinimide sodium salt) (NHS) (5 mg mL^−1^) in phosphate buffer saline (PBS) at pH 7.2. Finally, the resulting modified PSiO_2_ carriers were rinsed with PBS (pH 7.2) and dried under a nitrogen gas. Note that all reagents used for PSiO_2_ modification were obtained from Sigma-Aldrich Chemicals, Rehovot, Israel. Surface modification was verified using attenuated total reflectance Fourier transform infrared (ATR-FTIR) spectroscopy. Spectra were recorded using a Thermo 6700 FTIR instrument equipped with a Smart iTR diamond ATR device.

### 2.2. BMP-2 Loading and Release from PSiO_2_ Carriers

Bone morphogenetic protein-2 (BMP-2, Peprotech, Rehovot, Israel) was loaded into the PSiO_2_ carriers by physical adsorption and covalent attachment. In the physical adsorption approach, the protein loading was performed using the impregnation method [35,52]. The loading solution was prepared by dissolving 10 µg of BMP-2 in a 1:1 (*v*/*v*) solution of PBS and ddH_2_O to a final protein concentration of 20 µg mL^−1^. 20 μL of the latter solution were introduced onto the PSiO_2_ carrier (size of 7 mm × 3 mm) and allowed to infiltrate into the nanostructure for 2 h; after which, the solution was collected (for subsequent quantification of BMP-2 content). For covalent conjugation of the protein, the BMP-2 loading solution was introduced onto the amine-reactive NHS-activated PSiO_2_ carriers and allowed to infiltrate and react with the activated NHS ester groups for 2 h. Quantification of BMP-2 loading within the PSiO_2_ carriers was determined using BMP-2 ELISA kit (Peprotech, Rehovot, Israel) according to the manufacturer’s protocol. BMP-2 loading within PSiO_2_ carriers was determined by subtracting the measured protein content within the collected post-loading solution from that of the initial loading solution. BMP-2 loading efficacy was calculated by the following equation:

(1) $$BMP2\;loading\;efficacy\;\left[\%\right]=\frac{Weight\;of\;BMP2\;in\;PSiO_{2}\;carrier}{Weight\;of\;BMP2\;in\;loading\;solution}\times 100\;$$

In vitro BMP-2 release studies were performed by incubating the BMP-2-loaded PSiO_2_ carriers in 2 mL of PBS containing 1% bovine serum albumin fraction v (BSA) (MP Biomedicals, Irvine, CA, USA) and 0.02% sodium azide (Sigma-Aldrich Chemicals, Rehovot, Israel) at 37 °C. Every two days, aliquots were sampled and replaced with fresh PBS. The aliquots were then frozen in liquid nitrogen and stored at −20 °C until further analysis using the BMP-2 ELISA kit.

### 2.3. Isolation and Culture of Mesenchymal Stem Cells (MSCs) from Rabbit Bone Marrow (BM) 

The femur was isolated from a New Zealand rabbit, cleaned from soft tissues in Roselle Park Medical Institute (RPMI) medium (Biological Industries, Beit HaEmek, Israel), and sterilized by immersion in 70% ethanol for 1 min. Subsequently, the femur was rinsed with sterile PBS and the bone ends were separated using sterile scissors. Next, bone marrow (BM) cells were eluted from the bone by flushing them with a sterile syringe filled with RPMI medium. The cell suspension was centrifuged at 1400 rpm for 7 min and the supernatant was discarded. The cell pellet was resuspended in 21 mL of RPMI medium, followed by a subsequent centrifugation at 1400 rpm for 7 min. The supernatant was then discarded and the cell pellet was resuspended in 10 mL of RPMI medium supplemented with 10% fetal bovine serum (FBS), 1% *L*-glutamine, 1% penicillin (100 U mL^-1^) streptomycin (100 µg mL^−1^) solution, 50 µg mL^−1^ gentamicin (all reagent were supplied by Biological Industries, Beit HaEmek, Israel) and 0.01% β-mercaptoethanol (Sigma-Aldrich Chemicals, Rehovot, Israel). Next, the cell suspension was filtered through a 70-µm filter mesh (Corning, Corning, NY, USA) and counted by an automated cell counter (Countess II, Life Technologies, Waltham, MA, USA). The cells were then seeded into a T-75 culture flasks in a density of 6000 cells cm^-2^, cultured for 2 weeks and maintained in a humidified incubator at 37 °C containing 5% CO_2_. The medium was replaced every 3 days.

### 2.4. Cell Viability Assay

Alamar Blue™ assay (Thermo Fisher Scientific, Waltham, MA, USA) was used to study the cytotoxicity of the PSiO_2_ carriers on BM MSCs. Briefly, 5 × 10^5^ cells in 0.5 mL of RPMI medium were seeded in each well (24-well plates) and allowed to attach for 12 h, followed by several rinsing with 0.5 mL of PBS. Then, empty neat PSiO_2_ and amine-reactive NHS-activated PSiO_2_ carriers (termed in this work as empty chemically-modified PSiO_2_ carriers), BMP-2-loaded PSiO_2_ (via both physical adsorption and covalent conjugation) or a solution of free BMP-2 (50 ng mL^−1^) with 0.5 mL of fresh growth medium. After 1, 3 and 6 days, the wells were washed twice with 0.5 mL of PBS and cells were incubated in 10% (*v*/*v*) Alamar blue solution for 4 h at 37 °C with 5% CO_2_ and protected from light. Aliquots of 150 μL were sampled from each well and the fluorescence intensity was quantified using a microplate reader (Varioskan Flash, Thermo Scientific, Waltham, MA, USA), λ_ex_ = 535 nm and λ_em_ = 590 nm. Cell viability is expressed as the fluorescence intensity of experimental wells normalized to respective values of control wells (cells only).

### 2.5. In Vitro BMP-2 Bioactivity Assay

In vitro BMP-2 bioactivity assay was performed as follows: Second passage MSCs (5 × 10^5^ cells) were seeded in 0.5 mL of RPMI medium in each well (24-well plates) and allowed to attach for 12 h, followed by rinsing the wells twice with 0.5 mL of PBS. Subsequently, we introduced 0.5 mL of fresh growth medium containing the following different treatments: (i) empty neat PSiO_2_, (ii) empty chemically-modified PSiO_2_ carriers, (iii) BMP-2-loaded PSiO_2_ (via physical adsorption), (iv) BMP-2-loaded PSiO_2_ (via covalent conjugation), (v) free BMP-2 solution (10 ng mL^−1^), (vi) free BMP-2 solution (50 ng mL^−1^), (vii) free BMP-2 solution (100 ng mL^−1^) and (viii) control untreated cells. One PSiO_2_ sample was introduced in each well, and all experiments were done in triplicates. After 7 days, the PSiO_2_ samples and the growth medium were replaced with fresh medium containing new PSiO_2_ samples (either empty or BMP-2 loaded) or free BMP-2 solutions, as described above, and the cells were maintained for additional 7 days in a humidified incubator at 37 °C containing 5% CO_2_. After 14 days, ALP activity was evaluated by performing ALP staining using the ab242286 ALP staining kit (Abcam, Tel Aviv, Israel) according to the manufacturer’s protocol. Briefly, 14 days after induction of differentiation, the cells were washed with PBS containing 0.05% Tween-20 (Sigma-Aldrich Chemicals, Rehovot, Israel). Next, cells were fixed by incubation with the kit’s fixing solution for 2 min, followed by two washes with PBS containing 0.05% Tween-20. The cells were then incubated with the kit’s staining solution for 30 min and protected from light. Subsequently, the cells were washed twice with PBS and observed using an inverted light microscope (Nikon TE2000-S; Nikon Corporation, Japan and Leica DM18; Leica Microsystems, Wetzlar, Germany). Quantitative analysis of the positively-stained area in every image (*n* = 5 frames were taken for each well, all experiments were done in triplicates) was performed using the image processing program Fiji, a distribution of ImageJ (US National Institutes of Health, Bethesda, MD, USA). 

### 2.6. Integration of the PSiO_2_ Carriers into a 3D-Printed Scaffold for Critical Size Bone Defects 

A defect was created in a mandible of a New Zealand rabbit in accordance with the animal care and protection. Ethics approval was obtained from the RAMBAM Ethics Committee (approval number: IL0230218, 20 June 2018). The defect included the whole thickness of the bone and had a diameter of 10 mm to obtain a critical size defect. The implant was designed based on a computerized tomography (CT) scan of the defect in the animal using a Planmeca ProMax^®^ 3D Max (Planmeca, Helsinki, Finland). The dicom files were converted into stereolithography (STL) 3D files using Philips IntelliSpace Portal (Philips, Amsterdam, Netherlands). The scaffold was designed using the Freeform Computer-aided design (CAD) program (Rock Hill, SC, USA) and included a slot for the PSiO_2_ carriers in the center of the scaffold, where two PSiO_2_ samples were fixed facing different directions. The scaffold was then printed from polycaprolactone (PCL) using an Ultimaker 2+ printer (Ultimaker, Utrecht, Netherlands).

## 3. Results

### 3.1. Fabrication and Chemical Modifications of PSiO_2_ Carriers

**Figure 1:**
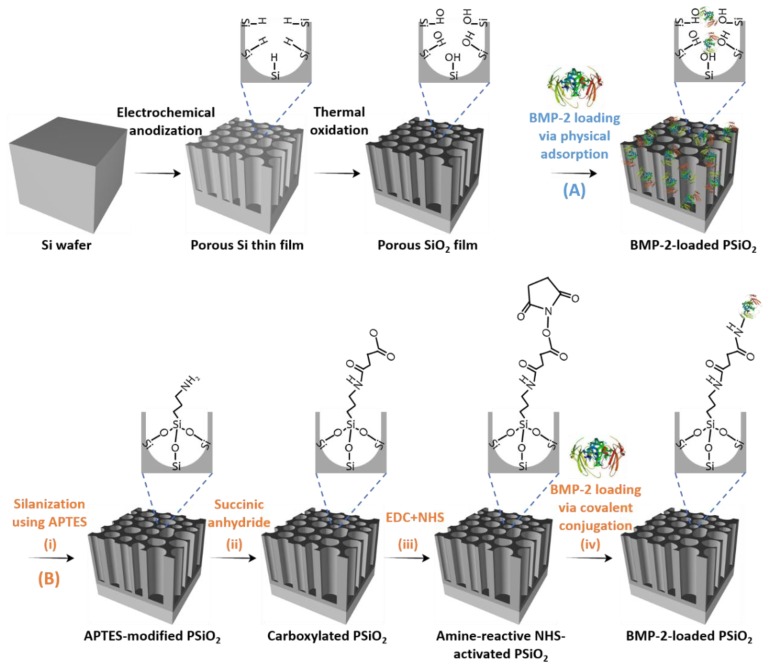
Schematic illustration of the fabrication of porous silicon (Psi) O_2_ carriers and subsequent bone morphogenetic protein (BMP)-2 loading via (**A**) Physical adsorption, or (**B**) Covalent attachment. A thin Si wafer is subjected to anodization at 250 mA cm^−2^ for 20 s, followed by thermal oxidation at 800 °C for 1 h to produce a PSiO_2_ scaffold. For conjugation of the protein to the carrier, the PSiO_2_ is modified using the following steps: (i) Reaction with (3-aminopropyl)triethoxysilane (APTES) to form an amine-terminated PSiO_2_; (ii) Introduction of succinic anhydride to yield a carboxylated surface; (iii) Carboxyl groups are activated into a reactive *N*-hydroxysulfosuccinimide sodium salt (NHS) ester intermediates by ethyl-3-(3-(dimethylamino)propyl)carbodiimide (EDC) and NHS; (iv) Conjugation of BMP-2 is carried out through reaction of its primary amines with the amine-reactive NHS esters (note that the schematics are not drawn to scale).

**Figure 2:**
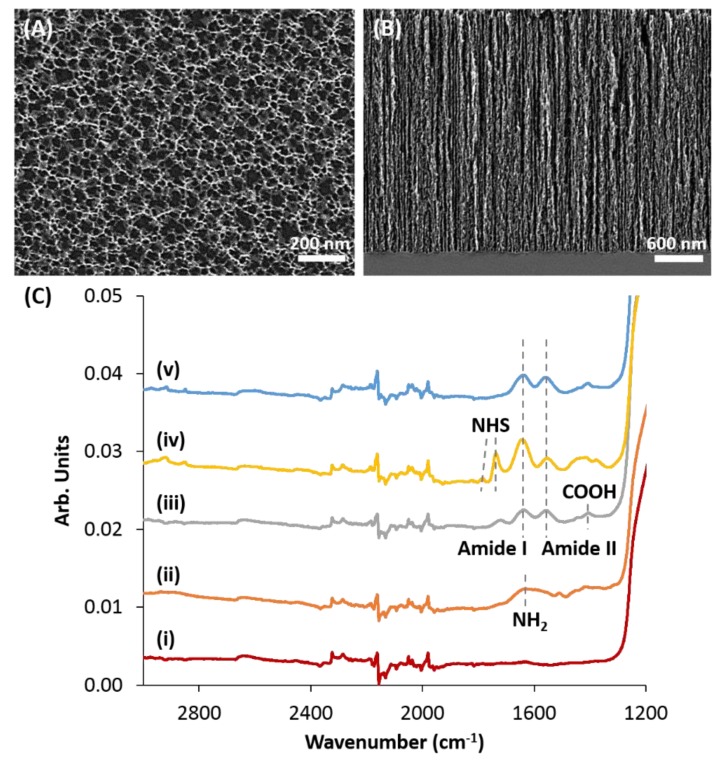
Characterization of the PSiO_2_ carriers by electron microscopy and ATR-FTIR spectroscopy. (**A**,**B**) Top-view and cross-section micrographs of a typical PSiO_2_ film etched at a current density of 250 mA cm^−2^ for 20 s. (**C**) ATR-FTIR spectra of PSiO_2_ following the different chemical modification steps performed for covalent conjugation of BMP-2: (i) Neat PSiO_2_; (ii) Amine-terminated surface after silanization with APTES; (iii) Carboxylated surface after modification with succinic anhydride; (iv) EDC/NHS activated surface; (v) BMP-2-conjugated PSiO_2_.

The PSi carriers were prepared by Si anodization at a constant current density of 250 mA cm^−2^ for 20 s, followed by thermal oxidation and dicing, as schematically illustrated in Figure 1. The anodization conditions were adjusted to efficiently accommodate the protein payload within the porous nanostructure, where BMP-2 has a molecular weight of 25.8 kDa and a diameter of ~4 nm [53,54]. The structure of the resulting oxidized PSi (PSiO_2_) films was characterized by HRSEM and their thickness was ~3 µm (Figure 2B) with a typical morphology of interconnecting cylindrical pores of approximately 40 nm in diameter (Figure 2A). The porosity of the films was determined by gravimetric studies [51], confirming their high porosity of ~77%. In this work, we studied two routes of protein loading; namely, physical adsorption and covalent attachment, as schematically illustrated in Figure 1. For the latter approach, the PSiO_2_ was first functionalized by amino-silanization (Figure 1Bi), which was followed by reaction with succinic anhydride. Subsequently, the BMP-2 protein was conjugated to the modified PSiO_2_ carriers via NHS and EDC coupling chemistry, see Figure 1B(iii,iv). 

The chemical functionalization on the PSiO_2_ surface was investigated using ATR-FTIR spectroscopy and the results are presented in Figure 2C. The neat PSiO_2_ exhibits a characteristic Si−H vibrating mode at 769 cm^−1^ and a peak at 1078 cm^−1^ that is attributed to the Si−O−Si stretching mode (data not shown). After the silanization step, a peak at 1626 cm^−1^ is observed, ascribed to the bending of the primary amines (Figure 2C(ii)) [51,55]. Following the modification with succinic anhydride, the spectrum shows two strong bands at 1551 and 1628 cm^−1^, see Figure 2C(iii), which are attributed to amide II and amide I bonds, respectively [51,55]. In addition, a peak at 1403 cm^−1^ is detected, assigned to the C−O stretching and O−H deformation vibrations of the carboxylic acid groups [56]. EDC/NHS coupling results in typical peaks at 1733 and 1778 cm^−1^, ascribed to the asymmetric and symmetric stretching bands of succinimidyl ester, respectively (Figure 2C(iv)) [51,55]. Following introduction of BMP-2, see Figure 2C(v), characteristic amide bands of proteins are observed at 1633 cm^−1^ (amide I) and 1553 cm^-1^ (amide II) [57].

### 3.2. BMP-2 Loading and Release from PSiO_2_ Carriers

**Figure 3:**
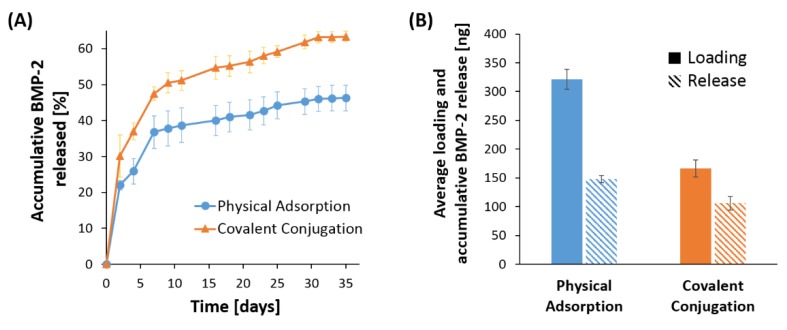
(**A**) BMP-2 release from PSiO_2_ carriers that were loaded by physical adsorption vs. covalent attachment of the protein. (**B**) Average BMP-2 loading and the corresponding accumulative release values for the two loading methods. Data represent mean ± SD, *n* = 3.

In this work, BMP-2 was loaded into the PSiO_2_ carriers by either physical adsorption or covalent attachment to the porous matrix. In the first method, the protein solution was allowed to infiltrate into the porous nanostructure and adsorption of the positively-charged BMP-2 molecules [3] to the negatively-charged PSiO_2_ surface is induced by favorable electrostatic interactions [36,38]. In the second approach, the BMP-2 protein was conjugated to the PSiO_2_ surface via EDC/NHS coupling chemistry, as illustrated in Figure 1B. Next, protein loading was quantified using a BMP-2 ELISA kit. Averaged protein content of 321 ± 18 ng and 167 ± 15 ng were achieved via physical adsorption and conjugation, respectively, corresponding to loading efficacy values of 82% and 43% (*w*/*w*). Release studies were performed in PBS (pH 7.4) at 37 °C under sink conditions, where every two days aliquots were sampled and replaced with fresh PBS and the amount of BMP-2 released from the carriers was quantified using a BMP-2 ELISA kit. Figure 3A presents the BMP-2 release profile throughout a period of 35 days from the loaded PSiO_2_ carriers in terms of accumulative percentage (see Figure S1 for the corresponding mass of released BMP-2). Both PSiO_2_ carriers exhibit a sustained release of BMP-2, without a burst effect, over a period of ~1 month, regardless of the loading method. Figure 3B presents the attained accumulative BMP-2 release values in comparison to the respective loading value for both loading methods. The release kinetics observed for both loading methods is similar throughout the studied period. Yet, when BMP-2 was loaded via physical adsorption, only 46% of the protein was found to be released over a one-month period; whereas, in the case of the conjugated protein, 63% of the loaded BMP-2 was released, see Figure 3B. This behavior is clearly observed from day 4 of the study (Figure 3A) and is consistent throughout the consecutive weeks. 

### 3.3. Cell Viability Studies for Proving the Biocompatibility of the PSiO_2_ Carriers.

**Figure 4:**
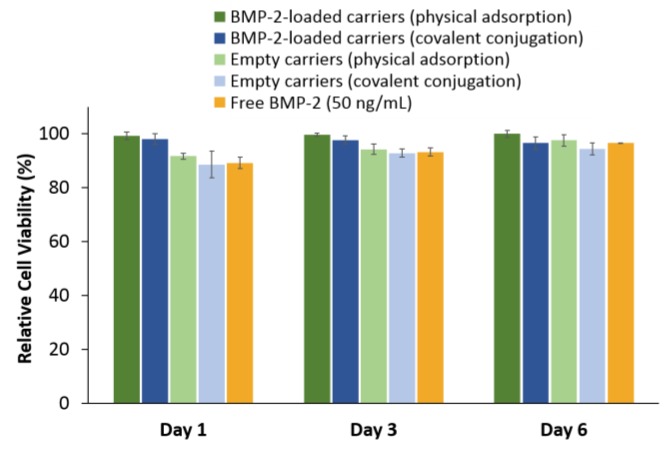
Cell viability of BM-MSCs grown with different PSiO_2_ carriers. The cells were cultured under one of the following conditions: (i) BMP-2-loaded PSiO_2_ carriers which were loaded via physical adsorption or covalent conjugation; (ii) Empty PSiO_2_ carriers (both neat PSiO_2_ and chemically-modified PSiO_2_ carriers); (iii) Supplementation of free BMP-2 solution at a concentration of 50 ng mL^−1^. Viability was tested on days 1, 3 and 6. The results are normalized to control untreated BM-MSCs and are presented as relative viability. Cell viability was determined by the Alamar Blue™ assay (*n* = 3).

The in vitro cytotoxic effect of the PSiO_2_ carriers was studied by their incubation with rabbit bone marrow mesenchymal stem cells (BM-MSCs). The cells were incubated with empty and BMP-2-loaded PSiO_2_ carriers, or supplemented with free BMP-2 (50 ng mL^−1^). Viability was quantified on days 1, 3 and 6 (post incubation) using the Alamar Blue™ assay and the results are summarized in Figure 4 and are normalized to cell viability values of the control untreated BM-MSCs. For all tested groups, the average relative cell viability was above 90% and no cytotoxic effect was observed. No significant differences were found between BM-MSCs cultured with neat and BMP-2-loaded PSiO_2_ carriers, as well as cells supplemented with free BMP-2 solution (as control). Thus, demonstrating the biocompatibility of the different carriers with the studied cells. It should be noted that previous studies suggested that resazurin-based viability assays (e.g., Presto Blue ™ and Alamar Blue™) may be incompatible with PSi due to possible non-specific reduction of the dye by the Si and its degradation products [58]. Therefore, to assure the assay reliability, the PSiO_2_ carriers were removed and the cells were thoroughly washed with PBS prior to introduction of the Alamar Blue™ solution [59].

### 3.4. Evaluation of the Bioactivity of the Released BMP-2

**Figure 5:**
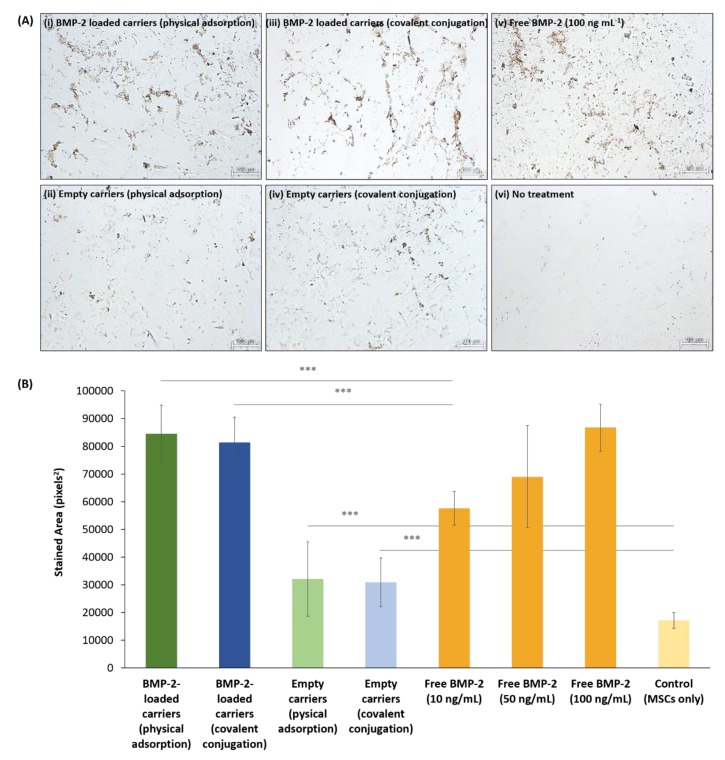
ALP staining of differentiated BM-MSCs after 14 days. (**A**) Representative light micrographs of cells exposed to (i) BMP-2-loaded PSiO_2_ carriers via physical adsorption, (ii) Empty PSiO_2_ carriers, (iii) BMP-2-loaded PSiO_2_ carriers via covalent conjugation, (iv) Empty chemically-modified PSiO_2_ carriers, (v) Free BMP-2 solution (100 ng mL^−1^) and (vi) no treatment (control, cells only). Scale bar = 100 µm. (**B**) Quantitative analysis of ALP activity, expressed as the average positively stained areas for each condition tested, *** indicates *p* ≤ 0.005.

To evaluate if the BMP-2 released from the PSiO_2_ carriers has retained its bioactivity, we examined its ability to induce osteogenic differentiation of BM-MSCs into osteoblasts in vitro compared to that of free BMP-2 supplementation. Two weeks after the exposure of BM-MSCs to BMP-2-loaded PSiO_2_ carriers or to different concentrations of free BMP-2 (10, 50, 100 ng mL^−1^), the bioactivity of BMP-2, using osteogenic differentiation assessment, was evaluated by monitoring the enzyme alkaline phosphatase (ALP) activity, which is considered as one of the most commonly used markers of osteogenesis [60,61]. Fourteen days after induction of differentiation, cells were ALP-stained and observed under a light microscope. Figure 5A shows representative micrographs of the stained cells grown in the presence of BMP-2 loaded PSiO_2_ carriers in comparison to BM-MSCs cultured with empty carriers or supplemented with free BMP-2 solution (control untreated cells are also presented for reference). A strong red staining is observed for both cells grown with the BMP-2 loaded carriers (Figure 5A-i,iii) and supplemented with free BMP-2 (Figure 5A-v). A negligible number of stained BM-MSCs are observed in the control untreated cells. Notably, stained BM-MSCs are also observed when grown with empty PSiO_2_ carriers (Figure 5A-ii,iv), suggesting some degree of osteogenic differentiation. Representative micrographs of the stained cells at a higher magnification are presented in Figure S2. 

Using image analysis software, the positively-stained areas were quantified and the results are summarized in Figure 5B. BM-MSCs cultured with BMP-2 loaded PSiO_2_ carriers (via both physical adsorption and covalent attachment) exhibit relatively high ALP activity, which is significantly higher than that achieved for treatment with free BMP-2 at concentration of 10 ng mL^−1^ (Figure 5B). The extent of ALP activity obtained with the loaded PSiO_2_ carriers was equipotent to that obtained with free BMP-2 treatment at concentrations of 50–100 ng mL^−1^. Namely, the BMP-2 released from the PSiO_2_ carriers was found to exhibit at least the same biological activity compared to that of free BMP-2 (or higher, depending on the applied free BMP-2 concentration), indicating that it has retained its bioactivity upon entrapment and release from the PSiO_2_ carriers. Importantly, MSCs cultured with empty PSiO_2_ carriers also demonstrated some extent of ALP activity, which was significantly higher than that of the control untreated cells (Figure 5B). 

### 3.5. Integration of the PSiO_2_ Carriers into a 3D-Printed Scaffold for Critical Size Bone Defects 

**Figure 6:**
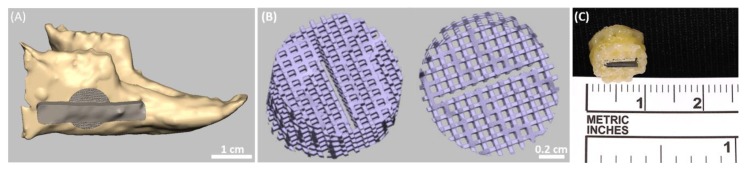
Design of the 3D printed scaffold for critical size bone defects. (**A**) 3D scanned rabbit mandible following bone resection and graft design using the freeform CAD program. (**B**) CAD design of the desired mesh (diameter of 10 mm) with an insert in its center to accommodate the PSiO_2_ carriers. (**C**) The 3D-printed PCL mesh containing the 7 mm × 3 mm PSiO_2_ carrier.

A nonhealing 10 mm full-thickness cylindrical defect was performed in a rabbit mandible. This defect removes both cortical plates and the intervening trabecular bone and tooth roots. Based on a CT scan of the mandible and using CAD software, a 3D model of the rabbit defected mandible was created. A customized graft, composed of a 10 mm round mesh, was designed, as shown in Figure 6A. The mesh is secured to the remaining bone using a solid bar (Figure 6A). Figure 6B presents the customized mesh graft, which is 10 mm in diameter and is comprised of square 500 µm pores and 300 µm-thick walls. An insert for the PSiO_2_ carriers (7 mm × 3 mm) is designed in the center of the mesh to accommodate two PSiO_2_ carriers facing opposite directions (Figure 6B). The customized graft was manufactured form poly(caprolactone) (PCL) by a 3D printer with a precision extruder deposition head (nozzle size 200 μm) system. Next, the PSiO_2_ carriers were incorporated within the PCL mesh and the integrated graft is shown in Figure 6C.

## 4. Discussion

### 4.1. BMP-2 Loading and Release from PSiO_2_ Carriers

The two most common approaches for loading molecular payloads into PSi carriers are physical adsorption or covalent attachment of the drug molecules to the Si scaffold using a variety of different convenient surface chemistries available [62,63,64]. In the latter method, the drug cargo is only released when the covalent bonds are broken or when the supporting PSi is degraded [65]. In this work, the loading of BMP-2 to the PSiO_2_ carriers was studied by both physical adsorption and covalent attachment to the porous matrix using silanization and carbodiimide coupling chemistry, as shown in Figure 1. For physical adsorption, as the BMP is positively-charged in aqueous media [3], electrostatic interactions between the protein and the negatively-charged PSiO_2_ surface are highly favorable for efficient loading [34,35,36,37,38,39]. When comparing the loading of BMP-2 using these two methods, we find that physical adsorption of the protein results in a significantly higher loading capacity compared to the covalent conjugation method (Figure 3B). In fact, the amount of BMP-2 entrapped within the carriers that were loaded via physical adsorption was almost double than the values achieved by covalent attachment. This behavior is attributed to the fundamental differences between these two methods. In covalent attachment, the cargo load is controlled by the density of the grafted linkers and the subsequent conjugation reaction yield within the nanostructured pores, which is also affected by hindered diffusion of the protein macromolecules. Moreover, the payload conjugation requires surface functionalization of the porous scaffold which in turn results in a reduced available pore volume [66]. Thus, the loading capacity (for a given loading solution) in this loading method is inevitably lower than that achieved by non-covalent loading techniques, such as the physical adsorption method [67,68,69]. In addition, possible denaturation of the sensitive protein molecules during conjugation can also account for the lower measured (quantified by ELISA) amount of BMP-2 loaded for the covalent attachment. Specifically, it is suggested that the abundance of primary amines in the side chain of lysine residues and the N-terminus of BMP-2, serving as targets for the NHS/EDC reagents, can lead to increased heterogeneity and restricted conformational flexibility due to multipoint attachment on the modified PSiO_2_ surface, favoring protein denaturation [70]. Importantly, the amount of loaded BMP-2 for both loading methods investigated in the present study correlates to clinically-relevant dose for in vitro induction of osteogenic differentiation [71,72,73]. 

The BMP-2-loaded PSiO_2_ carriers exhibit a sustained release of BMP-2 over a period of 35 days (Figure 3A). The prolonged release span reported here is longer than that achieved in other BMP-2 delivery systems that are based on silica nanotube meshes [74], carbon nanoparticles [75], composite poly(_L_-lactic-*co*-glycolic acid) and mesoporous silica membranes [76], as well as poly(_L_-lysine)/hyaluronan films [77]. As already pointed out, one of the main challenges in the delivery of BMP-2 is its rapid clearance rate [2,7]. Thus, the capability of the PSiO_2_ carriers to sustain the release of the protein for several weeks, while localizing its administration to the injured site, is highly advantageous. 

When comparing the release behavior from the loaded PSiO_2_ carriers, we found that carriers loaded via the covalent conjugation method exhibit higher percentage of released BMP-2, out of the total amount loaded, than that achieved via the physical adsorption method, see Figure 3B. This result may suggest that in the present case, the conjugation process actually stabilize the protein [78], while other effects such as the solubility of the protein conjugates (to Si moieties) cannot be excluded. 

### 4.2. Cell Viability and Bioactivity of the Released BMP-2

The in vitro cytotoxicity of the PSiO_2_ carriers, BMP-2-loaded and empty, was studied by their incubation with BM-MSCs cultures. These multipotent cells are the progenitor cells for osteoblasts, adipocytes, and chondrocytes and are widely used in the field of bone regeneration to study osteogenic differentiation [79,80,81]. The results presented in Figure 4 show no cytotoxic effect for both empty and BMP-2-loaded PSiO_2_ carriers. The viability of the BM-MSCs was comparable to that of the untreated cells, as well as to that of cells supplemented with free BMP-2 solution. Thus, confirming that the PSiO_2_ carriers are biocompatible with rabbit MSCs, in agreement with previous studies with these cells [29,82,83]. 

To confirm that the entrapment of BMP-2 within the PSiO_2_ carriers via either physical adsorption or covalent conjugation did not impede the bioactivity of the protein, we examined the ability of the released growth factor to induce osteogenic differentiation of rabbit BM-MSCs into osteoblasts compared to that of free BMP-2. It is well-established that when cultured in osteogenic media, MSCs tend to express markers which are characteristic of bone forming osteoblasts. BMPs are the most potent inducers of osteoblastic differentiation [84] and they are known to stimulate the primary signal for the differentiation of non-committed pluripotent cells into mineral-depositing osteoblasts [85]. Local administration of BMPs induces bone regeneration process. In vitro, osteogenic differentiation occurs throughout a period of 1 month. During the first two weeks, downregulation of DNA replication occurs and the cells begin to express osteoblast markers, mainly the enzyme ALP [86]. ALP plays an important role in the degradation of inorganic pyrophosphate, providing sufficient local concentration of phosphate or inorganic pyrophosphate required for mineralization. Therefore, ALP is commonly used as osteogenic differentiation marker, representing the degree of osteogenic differentiation [60,61]. Thus, we have exposed the BM-MSCs to the BMP-2 loaded PSiO_2_ carriers or to different free BMP-2 concentrations and ALP activity was measured 14 days later. We found that BMP-2 released from the PSiO_2_ carriers (regardless of the loading method) has retained its bioactivity and induced significant osteogenic differentiation, equivalent to that achieved by free BMP-2 solutions at concentrations of 50–100 ng mL^−1^, see Figure 5B. Remarkably, the empty PSiO_2_ carriers have also demonstrated some degree of differentiation, with an ALP activity which was significantly higher than the control untreated MSCs. We ascribe this behavior to the release of silicic acid moieties, during the erosion of the porous scaffold, into the culture media. Orthosilicic acid was already reported to stimulate osteoblast differentiation in vitro by upregulating microRNA-146a to antagonize NF-κB activation [33]. It has also been suggested that Si plays an important role in the expression of ALP [24]. In addition, Hing et al. [26]. have shown that incorporation of Si in porous hydroxyapatite implants is beneficial for early bone repair and new bone deposition. Thus, by enhancing osteogenic differentiation of MSCs, the combination of PSi and BMPs offers an interesting perspective for bone reparation and regeneration. 

### 4.3. Integration of the PSiO_2_ Carriers into a 3D-Printed Scaffold for Critical Size Bone Defects 

A critical size defect is an ideal model for testing bone regeneration platforms. Geometry of a 10 mm cylindrical defect was chosen for this study as it is easily accessible and reproducible [87]. A customized graft was designed to fit this critical size defect created in the rabbit mandible, comprised of square 500 µm pores and 300 µm-thick walls (Figure 6A). One of the most important characteristics of the scaffold is a high interconnected porosity to enable vascularization for nutrient and gas diffusion, which permits waste disposal [25,88,89]. Smaller pore sizes (< 200 µm) have been shown to result in a limited bone formation due to insufficient accessible pore volume, limited oxygen diffusion, and vascular invasion. A pore size of 500 µm is considered as an effective scaffold pore size which enables increased oxygen diffusion, pre-osteoblast cell infiltration, proliferation, and survival throughout the entire scaffold [90,91]. As the role of the PSiO_2_ is to provide a reservoir for the sustained delivery of the BMP-2 protein, we have localized the PSiO_2_ carriers in the center of the defect facing opposite directions (Figure 6C) to allow for optimal spatial distribution of the released protein in vivo. PCL was chosen as the printing material as it is a thermoplastic biocompatible material, with mechanical properties that resemble those of a trabecular bone, and thus would be sufficient to withstand the physiological forces of mastication. This polymer is characterized by a relatively slow degradation rate of 3–6 months [92,93,94,95] and thus can maintain physical integrity for primary stability in cases of large deficiencies. 

In summary, utilizing PSi-based delivery systems for local administration of osteoinductive growth factors and their integration within biodegradable osteoinductive implants opens up great possibilities for efficient bone-regeneration therapies. Our current work is focused on studying the therapeutic efficacy of the integrated grafts in vivo. 

## Supplementary Materials

The following are available online at https://www.mdpi.com/1999-4923/11/11/602/s1, Figure S1: BMP-2 release from PSiO_2_ carriers that were loaded by physical adsorption vs. covalent attachment of the protein, expressed as the accumulative BMP-2 mass released over time. Figure S2: ALP staining of differentiated BM-MSCs after 14 days. Representative light micrographs of cells exposed to (i) BMP-2-loaded PSiO_2_ carriers via physical adsorption, (ii) BMP-2-loaded PSiO_2_ carriers via covalent conjugation, (iii) Empty PSiO_2_ carriers, (iv) empty chemically-modified PSiO_2_ carriers, (v) Free BMP-2 solution (100 ng mL^−1^) and (vi) No treatment (control, cells only). Scale bar = 75 µm.
