# Supplementary material for: Bone Morphogenic Protein 2-Loaded Porous Silicon Carriers for Osteoinductive Implants
Source: Pharmaceutics. 2019 Nov 12;11(11):602. doi: 10.3390/pharmaceutics11110602 (PMC6920899; doi:10.3390/pharmaceutics11110602)
Supplement: Supplementary file 1 [file pharmaceutics-11-00602-s001.pdf]

pharmaceutics

Supplementary Materials: Bone Morphogenic Protein 2-Loaded Porous Silicon Carriers for Osteoinductive Implants

Michal Rosenberg, Dekel Shilo, Leonid Galperin, Tal Capucha, Karim Tarabieh, Adi Rachmiel and Ester Segal

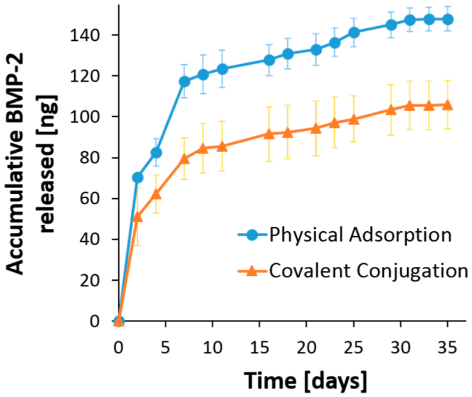

Figure 1. BMP-2 release from $PSiO_2$ carriers that were loaded by physical adsorption vs. covalent attachment of the protein, expressed as the accumulative BMP-2 mass released over time.

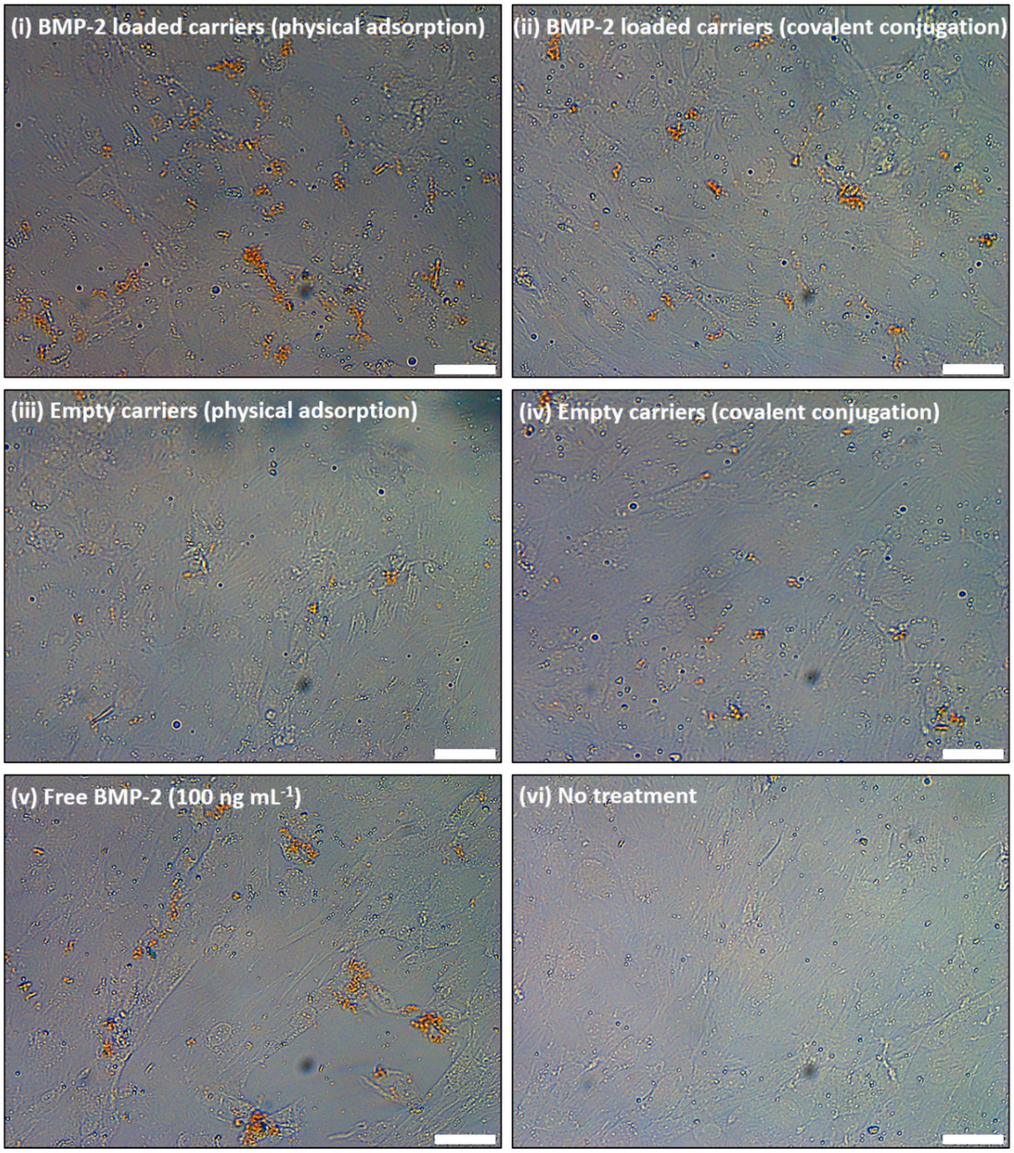

Figure S2. ALP staining of differentiated BM-MSCs after 14 days. Representative light micrographs of cells exposed to (**i**) BMP-2-loaded $PSiO_2$ carriers via physical adsorption, (**ii**) BMP-2-loaded $PSiO_2$ carriers via covalent conjugation, (**iii**) empty $PSiO_2$ carriers, (**iv**) empty chemically-modified $PSiO_2$ carriers, (**v**) free BMP-2 solution (100 ng mL^{-1}) and (**vi**) no treatment (control, cells only). Scale bar = 75 μm.
